# Epigenetic clocks galore: a new improved clock predicts age-acceleration in Hutchinson Gilford Progeria Syndrome patients

**DOI:** 10.18632/aging.101533

**Published:** 2018-08-21

**Authors:** Andrew E. Teschendorff

**Affiliations:** 1 CAS Key Laboratory of Computational Biology, CAS-MPG Partner Institute for Computational Biology, Shanghai Institute of Nutrition and Health, Shanghai Institute for Biological Sciences, University of Chinese Academy of Sciences, Chinese Academy of Sciences, Shanghai 200031, China; 2 UCL Cancer Institute, Paul O’Gorman Building, University College London, London WC1E 6BT, United Kingdom

**Keywords:** DNA methylation, epigenetics, epigenetic clock, aging, biological aging, progeria, fibroblast

**Comment on:** “Epigenetic clock for skin and blood cells applied to Hutchinson Gilford Progeria Syndrome and ex vivo studies” by Steve Horvath, et al. published in Aging Volume 10, Issue 7, pp 1758—75. http://www.aging-us.com/article/101508/text

Ever since in 2013 Steve Horvath showed that the chronological age of a healthy individual can be accurately predicted using a DNA methylation (DNAm) based predictor [1], and that deviations between the predicted and true age can be informative of lifespan and other age-related phenotypes [1,2], there has been a surge of follow-up studies deriving different types of more specialized epigenetic clocks aimed at measuring different aspects of the aging process. For instance, unlike Horvath’s clock, the “Levine clock” [3] was derived using biological measures of aging (e.g. measures of cognitive decline) and is therefore more tailored to measure biological aging in older individuals. Another example is a DNAm-based clock called “epiTOC”, aimed at approximating the mitotic age of a tissue, and which was shown to be more tailored for predicting cancer risk [4]. Although Horvath’s clock remains the only clock to accurately predict chronological age independently of tissue-type (hence also termed the “pan-tissue” clock), and to also predict age-acceleration in a wide range of different diseases [5], a long-standing puzzle has been its failure to predict age-acceleration in a disease like Hutchinson Gilford Progeria Syndrome (HGPS), a disease which exhibits all the hallmarks of age-acceleration [1,6].

In a recent paper published in the journal *Aging*, Horvath and colleagues offer a potential explanation and solution to this puzzle [7]. In the context of HGPS, a more relevant cell-type in which to measure DNAm are fibroblasts, and not immortalized lymphoblastoid cell-lines as done previously [1]. Interestingly, Horvath’s clock had been previously observed to not be well calibrated in fibroblasts, presumably because the associated samples had been cultured, while mostly *in-vivo* samples had been used in the training of the clock itself [1]. Thus, Horvath and colleagues set out to derive a new epigenetic clock for fibroblasts. An immediate challenge facing them however, was the fact that construction of an accurate clock requires on the order of many hundreds if not thousands of samples, while only a 100 fibroblast samples from four different studies were available. To overcome this problem, the authors used a larger training set consisting of approximately 900 samples, encompassing other tissues and cell-types, including buccal, blood and skin. Using the same statistical framework as in Horvath’s original work, the authors derived a new epigenetic clock for chronological age, consisting of 391 CpGs, called the “skin-blood clock’. Using this skin-blood clock, the authors applied it to fibroblast samples from the Progeria Research Foundation. This clock predicted age-acceleration in HGPS patients, despite the fact that the median age was only 8 years, a time when the rate of normal, i.e. healthy, epigenetic aging is fastest. Importantly, the association between epigenetic age-acceleration and HGPS status increased significantly after adjustment for passage number. This is an important finding, because, if validated in future larger studies, it provides a means of measuring epigenetic age in fibroblasts, which are easily isolated from an accessible tissue such as skin, and secondly, because it may allow *ex-vivo* monitoring of the success of progeria drug treatments.

The significance of the study however goes well beyond its implications for HGPS and other progeria syndromes. First, the skin-blood clock could be used for quantitative *ex-vivo* human cell aging assays generally, although its usefulness is likely to be restricted to age-related conditions and diseases where fibroblasts or other skin cell subtypes are relevant. Second, the authors applied the clock to cord blood, finding that it correlates with gestational age. Third, although the skin-blood clock was derived from significantly less samples (~900) than Horvath’s clock (~8000 samples), it was found to more accurately predict chronological age, not only across fibroblasts and skin, but also across blood, buccal and saliva tissue. A potential factor driving this improved accuracy in blood could be related to the approximate 18-fold increase in genomic coverage afforded by using Illumina 450k/850k beadarrays instead of the much older 27k beadarrays that were used in the derivation of the pan-tissue clock. Thus, the improved accuracy of the skin-blood clock makes it more suitable for forensic applications.

It should be noted that the skin-blood clock is not meant to be a replacement for Horvath’s pan-tissue clock. Although the skin-blood clock also predicts chronological age in other cell-types like neurons, glia and liver, it remains to be seen if the improved accuracy extends to the 30 or more tissue-types used in the validation of the pan-tissue clock. Moreover, the skin-blood clock is likely to still be biased towards a tissue like blood since over 70% of the training samples derived from whole blood, cord blood, peripheral blood mononuclear cells or from buccal swabs, a tissue which has recently been shown to contain a relatively high fraction of leukocytes [8]. Thus, looking forward, it would be important to design a study which constructs an epigenetic clock from a much larger number, say a thousand fibroblast samples, which would therefore not constitute a pan-tissue clock, but which would be more tailored towards measuring age-acceleration in HGPS patients. Such an improved tailored clock could form the basis for evaluating the effectiveness of progeria treatments via an *ex-vivo* assay. As such, the recently published study by Horvath and colleagues is highly significant as it serves as a roadmap for future clock studies, pointing towards the importance of constructing tissue or cell-type specific epigenetic clocks, to more accurately measure biological aging in the given tissue/cell-type, and therefore with the potential to be more informative of disease-risk or the success of disease interventions in the tissue or cell-type of interest. In doing so, studies should profile purified samples to avoid confounding by cell-type heterogeneity, or, if profiling complex tissues, must adjust for it using appropriate statistical methodology [8,9].

## References

[1] Horvath S. Genome Biol. 2013; 14:R115. 10.1186/gb-2013-14-10-r115 24138928 PMC4015143

[2] Marioni RE, et al. Genome Biol. 2015; 16:25. 10.1186/s13059-015-0584-6 25633388 PMC4350614

[3] Levine ME, et al. Aging (Albany NY). 2018; 10:573–91. 10.18632/aging.101414 29676998 PMC5940111

[4] Yang Z, et al. Genome Biol. 2016; 17:205. 10.1186/s13059-016-1064-3 27716309 PMC5046977

[5] Horvath S, Raj K. Nat Rev Genet. 2018; 19:371–84. 10.1038/s41576-018-0004-3 29643443

[6] Merideth MA, et al. N Engl J Med. 2008; 358:592–604. 10.1056/NEJMoa0706898 18256394 PMC2940940

[7] Horvath S, et al. Aging (Albany NY). 2018; 10:1758–75. 10.18632/aging.101508 30048243 PMC6075434

[8] Zheng SC, et al. Epigenomics. 2018; 10:925–40. 10.2217/epi-2018-0037 29693419

[9] Teschendorff AE, Relton CL. Nat Rev Genet. 2018; 19:129–47. 10.1038/nrg.2017.86 29129922

